# Tyrosine Hydroxylase–Positive Nucleus Accumbens Neurons Influence Delay Discounting in a Mouse T-Maze Task

**DOI:** 10.1523/ENEURO.0487-24.2024

**Published:** 2024-12-12

**Authors:** Ryan Appings, Justin J. Botterill, Mudi Zhao, Sadia Riaz, Asa Kanani, Francesca Violi, Carl Frank David Steininger, Rutsuko Ito, Maithe Arruda-Carvalho

**Affiliations:** ^1^Department of Psychology, University of Toronto Scarborough, Toronto M1C 1A4, Canada; ^2^Department of Anatomy, Physiology, and Pharmacology, University of Saskatchewan, Saskatoon, Saskatchewan S7N 5E5, Canada; ^3^Department of Cell and Systems Biology, University of Toronto Scarborough, Toronto M1C 1A4, Canada

**Keywords:** adolescence, chemogenetics, impulsive action, mouse, ventral tegmental area

## Abstract

Delay discounting (DD) is a phenomenon where individuals devalue a reward associated with a temporal delay, with the rate of devaluation being representative of impulsive-like behavior. Here, we first sought to develop and validate a mouse DD task to study brain circuits involved in DD decision-making within short developmental time windows, given widespread evidence of developmental regulation of impulse control and risk-taking. We optimized a T-maze DD task for mice that enables training and DD trials within 2 weeks. Mice learned to choose between a large and a small reward located at opposite arms of a T-maze. Once training criteria were met, mice underwent DD whereby the large reward choice was associated with a temporal delay. Task validation showed that adolescent C57BL/6J mice display an increased preference for the small reward upon a temporal delay, confirming increased impulsivity compared with adults. We next used this DD task to explore the neural basis of decision-making. We used tyrosine hydroxylase transgenic mice (TH-Cre) to target TH-positive neurons in the nucleus accumbens (NAc) and ventral tegmental area (VTA) with Cre-dependent excitatory or inhibitory designer receptors exclusively activated by designer drugs (DREADDs). Inhibition of transduced neurons in the NAc decreased preference for the small but immediate reward during DD. Inhibition of TH-positive neurons in the ventral tegmental area (VTA) did not affect impulsive choice in this DD task. These results uncover a novel role for NAc TH-positive neurons in DD behavior and expand the repertoire of behavioral tasks available for studying decision-making across the lifespan.

## Significance Statement

Delay discounting tasks are used in rodents to study impulsive choice, whereby subjects display a preference for an immediate, smaller reward when access to a larger reward is contingent on a temporal delay. Research implicates the nucleus accumbens (NAc) brain region in impulsive behavior, with recent evidence of specialization among NAc neuronal subtypes in impulsive choice. Here, we interrogated the neural requirements of impulsive choice in mice. We found that inhibition of a subset of NAc neurons expressing tyrosine hydroxylase decreases impulsive choice. We also saw increased impulsive choice in adolescent mice compared with adults, consistent with reported developmental changes in impulsivity. Together, our data identify cell-specific NAc regulation of impulsive choice with important implications for neurodevelopment.

## Introduction

Delay discounting (DD) refers to the subjective devaluation of a favorable outcome when there is a delay in experiencing it (Chung and Herrnstein, 1967; Ainslie, 1974). Discounting rates capture the degree of devaluation as the delay in receiving a reward increases, with steeper rates representing more impulsive-like behavior (Ainslie, 1975). Adolescence is characterized by an increased tendency toward risk-taking behaviors (Arnett, 1992; Spear, 2000a; Blakemore and Robbins, 2012) that coincides with developmental deficits in impulse control that can present as one of two forms: impulsive action, wherein an individual is incapable of inhibiting a prepotent motor response, or impulsive choice, when a less beneficial reward is favored over a more gratifying one due to associated contingencies [e.g., delay or probability (Romer et al., 2017)]. In humans, adolescents exhibit steeper DD rates than adults (Green et al., 1994; Steinberg et al., 2009; D. C. Lee et al., 2015; Van Den Bos et al., 2015; but see Scheres et al., 2014). Similarly, adolescent rodents generally show heightened impulsive choice, preferring an immediate small reward over a delayed large reward compared with adults (Adriani and Laviola, 2003; Pinkston and Lamb, 2011; Doremus-Fitzwater et al., 2012; McClure et al., 2014; Lukkes et al., 2016). Importantly, 75% of all mental disorders emerge in adolescence (Kessler et al., 2005), of which major depressive disorder, schizophrenia, bipolar disorder, and bulimia nervosa (Kessler et al., 2005) have been associated with steeper DD (Amlung et al., 2019). Furthermore, initiation of substance use is also prevalent in adolescence (Blanco et al., 2018), and steeper DD is correlated with substance use (Vuchinich and Simpson, 1998), dependence (Madden et al., 1997; Bickel et al., 1999; Coffey et al., 2003), and addiction severity (Amlung et al., 2017), highlighting the importance of studying adolescent DD.

Human and animal studies implicate the mesocorticolimbic circuit in regulating impulsive behavior (Probst and Van Eimeren, 2013), with an important role for adolescent dopaminergic signaling in guiding adult behavior (Naneix et al., 2013; Reynolds et al., 2019; Reynolds and Flores, 2021). In particular, rodent lesion studies implicate the nucleus accumbens (NAc) in impulsive choice (Cardinal et al., 2001; Acheson et al., 2006; Bezzina et al., 2007; da Costa Araújo et al., 2009; Basar et al., 2010; Galtress and Kirkpatrick, 2010; Valencia-Torres et al., 2012; Feja et al., 2014; Steele et al., 2018). Furthermore, while some studies show a role for NAc dopamine signaling in modulating decision-making in DD (Saddoris et al., 2015; Orsini et al., 2017), depletion of NAc dopamine does not affect impulsive choice (Winstanley et al., 2005), suggesting further work is necessary to clarify the contribution of NAc dopamine signaling to DD. The NAc is predominantly comprised of GABAergic medium spiny neurons (MSNs) displaying diverse colabeling with markers such as calbindin, neuropeptides, and tyrosine hydroxylase (TH; Tashiro et al., 1990; Meredith, 1999; Basar et al., 2010). While there is evidence for specialization in the NAc neurochemical regulation of DD behavior (Yates and Bardo, 2017), the contribution of different NAc neuronal cell types to impulsive choice remains underexplored.

Here, we leveraged a DD task capable of capturing changes in impulsive choice within short developmental windows in mice to explore the contribution of neuronal subpopulations to impulsive choice. In the first part of this study, we adapted a T-maze task conventionally used in adult rats (Olmstead et al., 2006) to measure DD in C57BL/6J mice. We validated our task by showing that adolescent mice display an increased preference for an immediate small reward over a delayed large reward compared with adults, a measure of increased impulsivity. In the second part of this study, we leveraged this DD T-maze task to explore the neural basis of impulsive choice in adult mice by modulating neural activity in the NAc and ventral tegmental area (VTA) in a cell-type–specific manner. Several studies have identified a discrete population of TH-positive neurons in the NAc (Voorn et al., 1986; Tashiro et al., 1990; Mura et al., 1995; K. Ikemoto et al., 1996, 1998; Cossette et al., 2005a; Björklund and Dunnett, 2007; Ugrumov, 2009; Bupesh et al., 2014; Depboylu et al., 2014; R. Chen et al., 2021), whose behavioral function is unknown. Thus, to attempt to clarify the contribution of dopaminergic mesolimbic signaling on DD behavior and probe the function of this understudied NAc neuronal cell type, we focused our manipulations on TH-positive neurons in both NAc and VTA. Chemogenetic inhibition of NAc TH-positive neurons decreased impulsivity, while bidirectional manipulation of VTA TH-positive neurons did not affect impulsive choice. Our findings expand the behavioral toolbox for studying decision-making behaviors in mice and highlight a novel role for TH-positive NAc neurons in modulating impulsive choice.

## Materials and Methods

### Animals

Female and male C57BL/6J mice (The Jackson Laboratory) were bred at our local facility. TH-Cre mice (Lindeberg et al., 2004; Botterill et al., 2021) were kindly provided by Dr. Jonathan Britt (McGill University) and subsequently bred in-house. Animals were the progeny of F1 and F2 breeders, and the date of birth was designated as postnatal day (P)0. Pups were kept with the dam until weaning at P21 and subsequently housed 2–5 per cage on a 12 h light/dark cycle (lights on at 07:00 h) with food and water delivered *ad libitum* until the time of the study. For the experiments comparing adolescent and adult animals, litters were divided by sex, and males and females were evenly distributed to either the adolescent or adult groups. For the experiments manipulating NAc and VTA activity, adult (P90 or older) mice were used. All behavioral procedures were performed daily during the light cycle under dim illumination. All animal care and manipulations were approved by the University Animal Care Committee of the University of Toronto.

### Drugs

The DREADD agonist compound 21 (C21; #HB6124, Hello Bio) was dissolved in 0.9% NaCl physiological saline at 0.5 mg per ml, with 1 ml aliquots kept frozen (−20c) until the day of experiments. C21 was injected (2 mg/kg, i.p.) 1 h before the start of DD sessions on Days 5 and 6 using a 0.3 ml insulin syringe. To account for any potential off-target effects of C21, we opted for a within-subject design in which all animals underwent C21 treatment.

### Food restriction

Food restriction began at the start of habituation on the T-maze. For the experiments comparing adolescent and adult animals, mice in the adolescent and adult groups were placed on food restriction starting at P23 and P49, respectively. Animals’ baseline free-feeding weights were recorded at the end of the day, and food was subsequently taken away from the hopper. A tablespoon of sucrose pellets was sprinkled onto the cage floor for mice to explore and eat overnight. For the remainder of the study, mice were weighed prior to the T-maze sessions, and food was added to their hopper approximately 20 min following the behavioral experiments. Mice were kept at approximately 85% of their free-feeding weight.

### Behavioral experiments

#### Behavioral apparatus

A plexiglass T-maze (45 × 10 × 10 cm arms, with a 10 × 10 × 10 cm central hub, 93 cm from the floor) was used for all experiments. A fourth, northern arm of the plus maze (45 × 10 × 10 cm that branched off at 90° angles from a 10 × 10 × 10 cm central hub) was blocked off using a square panel, thereby creating the T-maze. A Basler Ace Classic camera was placed 108 cm above the T-maze. The testing room was dimly lit (200 lm) and had white noise playing in the background. The walls surrounding the T-maze apparatus were plastered with different pattern wallpapers acting as extramaze visual cues. Between animals, the maze and empty holding cage were cleaned with 70% ethanol.

#### Handling and habituation

For handling and all behavioral procedures, animals were transported in their homecage to the testing room. Animals were handled on the palm of the hand for two 2 min sessions across 5 consecutive days, once in the morning and once in the afternoon. On the fifth day, animals in the same cage were placed into the T-maze for a group habituation session of 5 min following each handling session. For the experiments comparing adolescent and adult animals, handling began at P21 and P47 for the adolescent and adult groups, respectively. These ages were strategically selected so that DD and reversal learning would be measured during adolescence (approximately P30–P45) or early adulthood (approximately P55–P70; Spear, 2000a).

#### Forced trials and training

Animals were trained on the T-maze to distinguish between two reward arms. The southernmost part of the T-maze served as the start arm. The two opposing east and west arms served as the reward arms, at the end of which were small metal bowls (1 cm diameter) containing either a small (one sucrose pellet; 5 mg, test diet) or large (six sucrose pellets) reward. To learn the difference between the two reward arms, animals initially underwent forced trials, followed by training.

##### Forced trials

The day after group habituation, mice were individually placed into the T-maze and allowed to freely explore the apparatus until they discovered and ate one sucrose pellet from each reward arm. They were subsequently assigned a large reward arm (counterbalanced across groups) and introduced to 10 forced trials, wherein one of the reward arms was closed off, forcing the animal into the other. For the NAc and VTA manipulations, sucrose pellets were only introduced to mice after the reward arms were assigned in the forced trials. A list randomizer was used to create a randomized order of trials, such that animals sampled both the large reward arm (six sucrose pellets) and small reward arm (one sucrose pellet) five times each. A trial ended when the animal had finished eating all the sucrose pellets from the bowl, or after 5 min of entering the reward arm. Between trials, mice were placed in an empty holding cage for a 1 min intertrial interval. After the 10 forced trials, animals were immediately returned to their homecage. Between animals, the maze and empty holding cage were cleaned with 70% ethanol.

##### Choice trials

Starting on the following day (Day 7 from the start of handling), animals underwent one training session (10 choice trials) per day until criterion performance was reached. During choice trial sessions, mice were placed into the southernmost starting point facing away from the rest of the T-maze and allowed to freely enter either the east or west arms. Once an animal had picked a (large or small) reward arm, with all four paws entering the arm, an opaque square panel was used to confine that animal into the chosen arm. This was done to ensure that mice learned that once they had entered an arm, they could only retrieve the reward that was in that arm. A trial ended when the animal had finished eating all the sucrose pellets from the bowl, or after 5 min of entering the reward arm. Between trials, mice were placed in an empty holding cage for a 1 min intertrial interval. After 10 trials, animals were immediately returned to their homecage. Animals underwent one choice trial session per day at the same hour (±1.5 h) until they reached the learning criterion, which was defined as entering the large reward arm for 8 out of 10 trials for 2 consecutive training days, measuring up to 80% accuracy.

#### Delay discounting

Once an animal had reached the learning criterion, they entered the DD phase on the following day, irrespective of the performances of their cage mates. Mice were assigned to either a 5 or 10 s delay depending on the experimental design. During DD, a transparent barrier was added directly in front of the sucrose bowl exclusively in the large reward arm. Similar to choice trials, animals were free to choose which reward arm to enter and were locked in once all four paws were inside the chosen arm. However, if they chose the large reward, they were locked between the start of the arm and the transparent barrier (being able to see but without access to the sucrose pellets) for a delay of 5 or 10 s. The delay period began as soon as an animal's nose reached the transparent barrier. After the delay, the transparent barrier was removed, and animals were allowed to retrieve the sucrose pellets. Each DD day consisted of 10 trials, and animals underwent the DD phase for 6 consecutive days. The percent choice of large reward was calculated as the percent entry into the large reward arm averaged across DD Days 5 and 6.

#### Reversal learning

Animals were trained on the T-maze to distinguish between two reward arms (as previously described). Once they met the learning criterion, the two reward arms were swapped, such that the arm that once contained the small reward now contained the large reward and vice versa. Mice then underwent choice trial sessions until they met the learning criterion for the new reversed framework.

### Stereotaxic surgery and viral injections

TH-Cre mice underwent stereotaxic surgery between 2 and 3 months of age. Briefly, mice were injected intraperitoneally (i.p.) with a combination of ketamine (100 mg/kg) and xylazine (5 mg/kg) to induce anesthesia. Once anesthetized, the head was shaved and swabbed with iodine followed by 70% ethanol. Tear gel (Alcon) was applied to the eyes to prevent dehydration. Mice were then secured in a rodent stereotaxic apparatus (Stoelting) using ear bars. Body temperature was maintained throughout surgery with a heating blanket. An incision was made down the midline of the scalp using a scalpel, the connective tissue was excised, and then the skull was cleaned with sterile phosphate-buffered saline (PBS, pH = 7.4). An autoclaved cotton-tip applicator was briefly submerged in 30% hydrogen peroxide and gently applied to the skull surface to identify the bregma. Using the bregma as a reference point, craniotomies were made bilaterally over the NAc (+1.5 mm anteroposterior, ±0.85 mm mediolateral) or ventral tegmental area (VTA; −3.15 mm anteroposterior, +/− 0.45 mm mediolateral).

The virus was delivered using a 500 nl Neuros syringe (#65457-02, Hamilton Company) attached to the stereotaxic apparatus with a probe holder (#751873, Harvard Apparatus). The syringe was positioned above each craniotomy, and the needle was lowered 4.5 mm below the skull surface to reach the NAc or VTA. For each injection, 0.2 µl of virus was injected at a rate of 0.06 µl/min. The following viral constructs were used: AAV5-hSyn-DIO-mCherry (≥7 × 10^12^ vg/ml, Addgene #50459), AAV5-hSyn-DIO-hM3D(Gq)-mCherry (≥7 × 10^12 ^vg/ml, Addgene #44361), or AAV5-hSyn-DIO-hM4D(Gi)-mCherry (≥7 × 10^12 ^vg/ml, Addgene #44362). The needle remained in place for an additional 5 min after each injection to allow for diffusion of the virus, and then it was slowly removed from the brain. Ketoprofen (1 mg/kg, s.c.) was injected approximately 30 min prior to the end of surgery to reduce discomfort. The skull was cleaned with sterile PBS, and the scalp was sutured with Vetbond tissue adhesive (3M). Mice were injected with 0.7 ml of warmed physiological saline at the end of surgery to support hydration. Mice were then transferred into a clean cage located on a heating blanket. Mice were returned to their colony room once fully ambulatory. Ketoprofen (1 mg/kg, s.c.) was administered 24 and 48 h after surgery to reduce postsurgical discomfort.

### Statistical analyses

**Figure 1.**
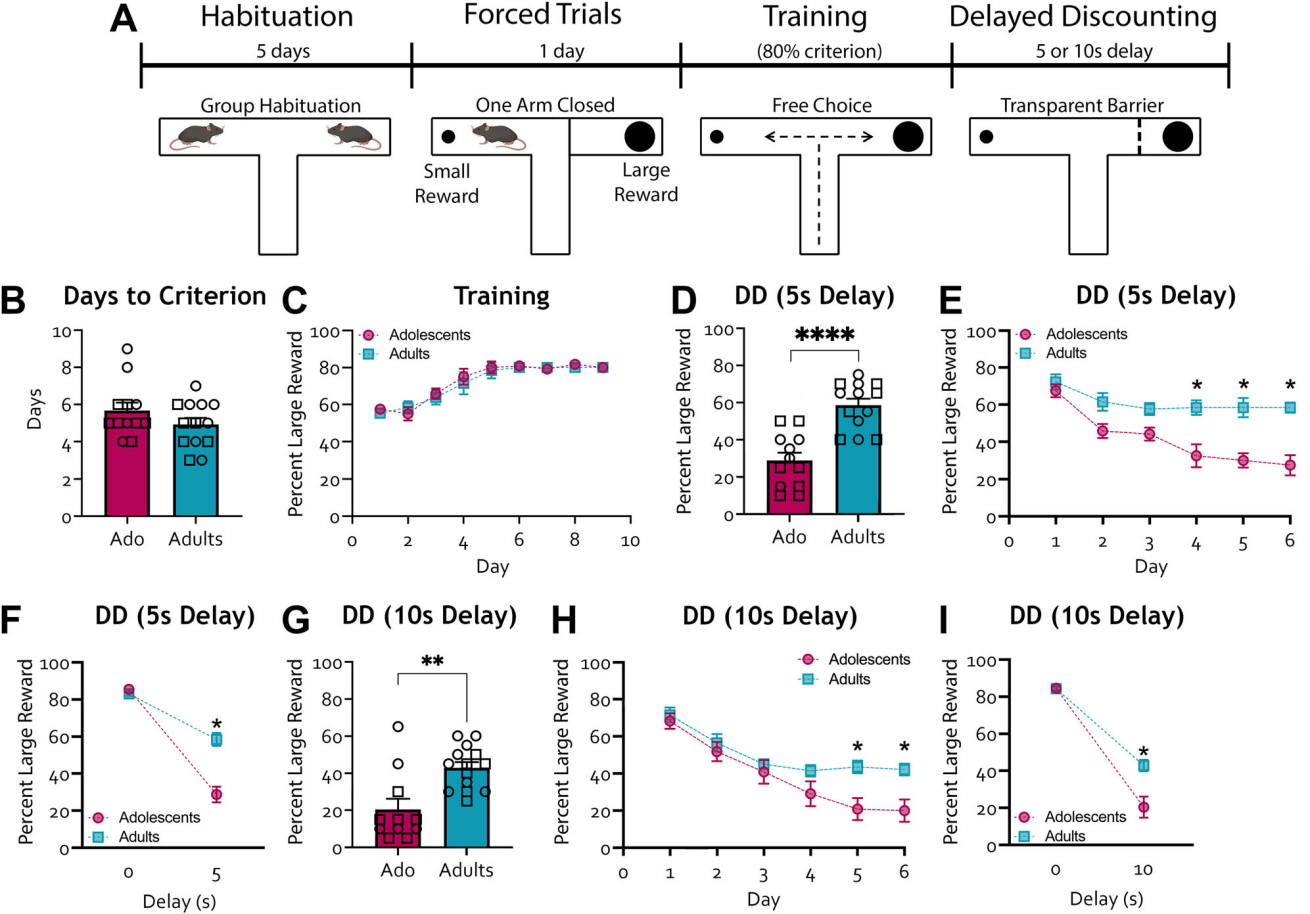
Delay discounting in C57BL/6J adolescent and adult mice. ***A***, Schematic of the experiment. The T-maze task is divided into four stages: handling and habituation, forced trials, training, and delay discounting (DD). Animals were trained to enter the large reward arm (counterbalanced between the left and right T-maze arms across mice) in 8 out of 10 trials across 2 consecutive training days. A 5 or 10 s delay contingency was then added to retrieving the sucrose from the large reward arm, and decision-making was assessed for 6 d. ***B***, Number of days to reach the learning criterion (at least 80% preference for large reward for 2 consecutive training days) did not differ between age groups (*t*_23_ = 1.349, *p* = 0.1904). Adolescents, *n* = 12 (5 females, 7 males); adults, *n* = 13 (7 females, 6 males). ***C***, Percent choice of large reward during training days did not differ between adolescents and adults (two-way ANOVA found no main effect of age, *F*_(1, 23)_ = 0.2371, *p* = 0.6309, and no age by training day interaction, *F*_(8, 184)_ = 0.2628, *p* = 0.9769). There was a significant main effect of time as animals learned the difference between large and small reward arms over the training. If an animal had already reached the criterion, the value for additional training days was set to 80% to avoid misleading dips in the acquisition curve. Adolescents, *n* = 12 (5 females, 7 males); adults, *n* = 13 (7 females, 6 males). ***D***, Percent choice of large reward with a 5 s delay contingency for the large reward was significantly lower in adolescents compared with adults. Adolescents, *n* = 12 (5 females, 7 males); adults, *n* = 13 (7 females, 6 males). ***E***, Percent choice of large reward with a 5 s delay contingency for the large reward was significantly lower in adolescents compared with adults at DD Days 4, 5, and 6 (Bonferroni's multiple-comparisons test, adolescents vs adults Day 1, *p* > 0.9999; Day 2, *p* = 1.033; Day 3, *p* = 0.0612). ***F***, Percent choice of large reward was reduced in both age groups after the addition of a 5 s delay. However, it reduced significantly more in adolescents than adults compared with the absence of delay (Bonferroni's multiple-comparisons test, no-delay adolescents vs adults, *p* > 0.9999). ***G***, Percent choice of large reward was lower in adolescents than adults with a 10 s delay contingency. Adolescents, *n* = 11 (5 females, 6 males); adult, *n* = 14 (8 females, 6 males). ***H***, Percent choice of large reward with a 10 s delay contingency was lower in adolescents compared with adults at DD Days 5 and 6 (Bonferroni's multiple-comparisons test, adoloscents vs adult Day 1, *p* > 0.9999; Day 2, *p* > 0.9999; Day 3, *p* > 0.9999; Day 4, *p* > 0.3682). There was also a significant main effect of time as animals learned the difference between large and small reward arms over the training. ***I***, Percent choice of large reward reduced in both age groups after the addition of a 10 s delay. However, it reduced significantly more in adolescents than adults compared with the absence of delay (Bonferroni's multiple-comparisons test, no-delay adolescents vs adults, *p* > 0.9999). *p < 0.05, **p < 0.01. Each data point is represented as a square (male) or a circle (female) for transparency.

**Figure 2.**
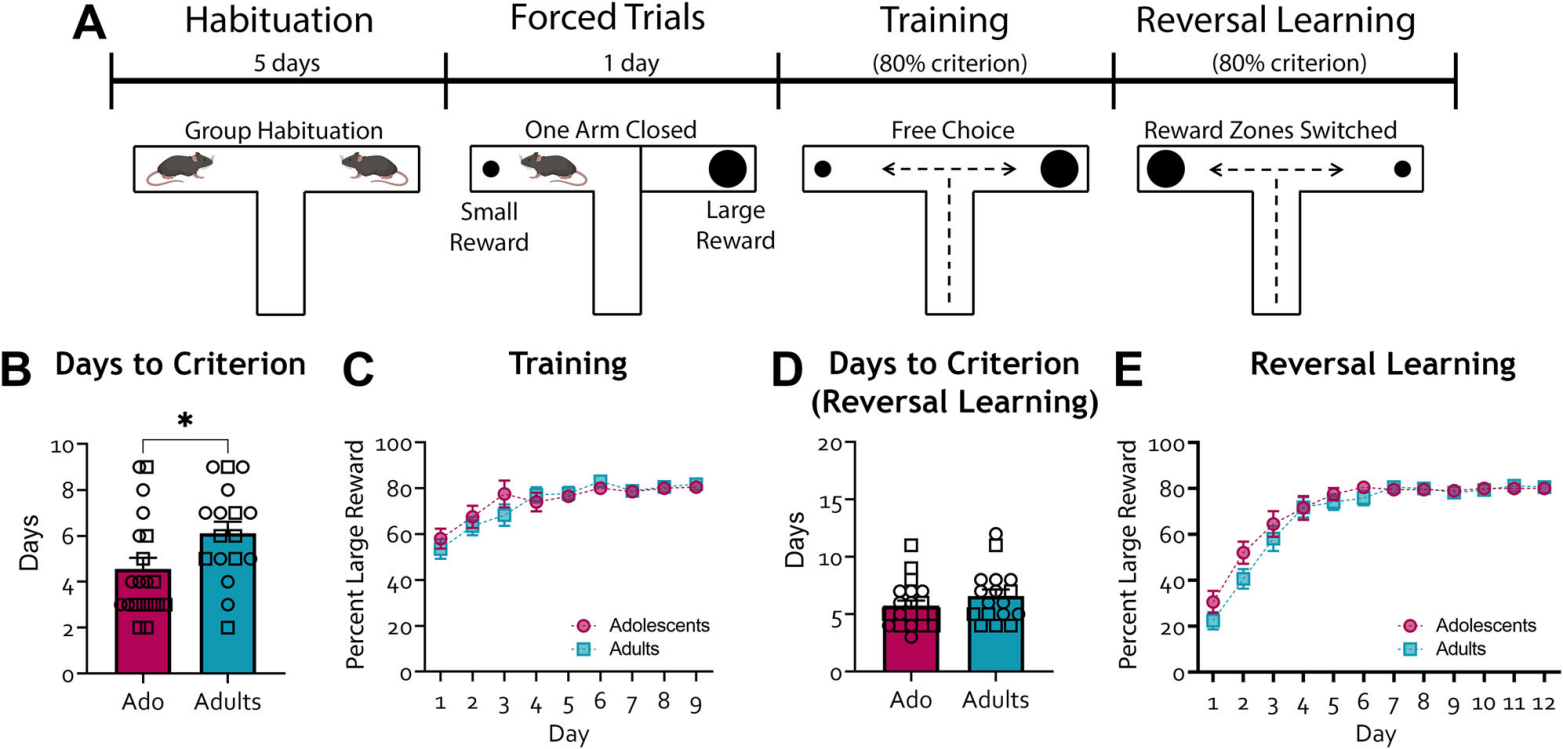
Reversal learning in C57BL6/J adolescent and adult mice. ***A***, Schematic of the experiment. Animals were trained to enter the large reward arm (counterbalanced across mice) in 8 out of 10 trials across 2 consecutive training days. The large and small reward arms were subsequently reversed, and animals were trained until they met the learning criterion for the new large reward arm. ***B***, Number of days to reach the learning criterion (at least 80% preference for large reward over 2 consecutive training days) was significantly lower in adolescents compared with adults. ***C***, Percent choice of large reward during training days did not differ between adults and adolescents (two-way ANOVA found no main effect of age, *F*_(1, 35)_ = 0.1744, *p* = 0.6788, and no age by training day interaction, *F*_(8, 280)_ = 0.8924, *p* = 0.5232). There was a significant main effect of training day as food-restricted animals with free choice learned the location and had an increased preference for the large reward. ***D***, Number of days to reach the learning criterion with the reversed framework (80% preference for new large reward arm over 2 consecutive training days) did not differ between adolescents and adults (*t*_35_ = 1.189, *p* = 0.2425). ***E***, Percent choice of large reward across reversal learning days did not differ between groups (two-way ANOVA found no main effect of age, *F*_(1, 35)_ = 1.280, *p* = 0.2656, and no age by training day interaction, *F*_(11, 385)_ = 1.056, *p* = 0.3958). Similar to the choice trials phase, there was a significant main effect of reversal learning day as animals learned the new location of the large reward. If an animal had already reached the criterion, the value for additional reversal learning days was set to 80% to avoid misleading dips in the acquisition curve. Adolescents, *n* = 20 (9 females, 11 males); adults, *n* = 17 (11 females, 7 males). From the adult group, five animals were excluded because they did not reach the learning criterion by the 9th training day, and one animal was excluded from the adolescent group. *p < 0.05, **p < 0.01. Each data point is represented as a square (male) or a circle (female) for transparency.

Data are presented as mean ± SEM. All statistical analyses were obtained using GraphPad Prism software version 9 with significance indicated at *p* < 0.05. For Figures 1 and 2, two-way ANOVA tests were conducted to compare adolescent and adult group means in each experimental condition. Since we found no statistically significant effect of sex on acquisition or preference for reward, female and male data were pooled within each group, and unpaired *t* tests were performed to minimize the chance of Type II errors. When examining learning curves, to account for missing values due to variance in the number of days required for an animal to reach the learning criterion, a value of 80% was arbitrarily inputted for animals across additional choice trial days, assuming that if they picked the large reward arm 80% of trials over 2 consecutive choice trial days, they would continue to exhibit this preference if no parameters of the task changed. Two-way repeated measures ANOVA (rmANOVA) were conducted to examine the trajectory of large and small reward arm entries across training and DD days. Bonferroni’s multiple-comparisons post hoc analyses were used over Sidak comparisons to avoid Type I errors.

10.1523/ENEURO.0487-24.2024.f1-1 Figure 1-1 **Optimization of the T maze DD task. A.** Schematic of experiment. The T-maze is comprised of three arms: the starting arm and two reward arms, containing a different amount of sucrose pellets. The task is broken down into four stages: handling and habituation, forced trials, training, and delay discounting (DD). Food restricted animals underwent a training phase and learned the difference between a large and small reward arm (counterbalanced between the left and right T-maze arms across mice). Training phase ended when animals reached criterion of a preference for the large reward in 8 out of 10 trials across 2 consecutive days. Next, in the DD phase (6 days), a 5 or 10 second delay contingency was added for the large reward arm. **B.** Quantification of the number of animals that reached the learning criterion (grey) or failed to complete training (white). Pilot 1 had 40% of animals reach learning criterion, Pilot 2 had 11.1% and the final DD paradigm had 100% **C.** Table outlining parameters used for each pilot and the final optimized DD paradigm. Download Figure 1-1, TIF file.

10.1523/ENEURO.0487-24.2024.f1-2 Figure 1-2 **Relationship between percent free-feeding weight and choice of small reward in C57BLK6/J adolescent and adult mice. A.** Percent free-feeding weight averaged across delay days 5 and 6 did not differ between the adolescent and adult groups (*t_23_* = 2.001, *p* = 0.0574). **B**. Simple linear regression of an animal’s percent free-feeding weight averaged across delay days 5 and 6 and the percent choice of large reward with a 5  s delay contingency (*ß* = -0.9312, *F_1, 23_* = 0.2662, *p* = 0.6108, *R*^2^ = 0.01144). **C*.*** Percent free-feeding weight averaged across delay days 5 and 6 did not differ between the adolescent and adult groups (*t_23_* = 1.440, *p* = 0.1634). **D**. Simple linear regression of an animal’s percent free-feeding weight averaged across delay days 5 and 6 and the percent choice of large reward with a 10  s delay contingency (*ß* = -0.3644, *F_1, 23_* = 0.1229, *p* = 0.7291, *R*^2^ = 0.005314). Adolescents, *n* = 12 (5 females, 7 males); adults, *n* = 13 (7 females, 6 males). **E-F**. Simple linear regression of animal weight averaged across delay days 5 and 6 and the percent choice of large reward for adolescent (**E**: *p* = 0.2145, *R*^2^ = 0.07237) and adult (**F**: *p* = 0.8173, *R*^2^ = 0.002175) mice. Adolescents, *n* = 15 (7 females, 8 males); adults, *n* = 17 (8 females, 9 males). *p *<* 0.05, ** *p* *<* *0.01.* Each data point is represented as a square (male) or a circle (female) for transparency. Download Figure 1-2, TIF file.

**Figure 3.**
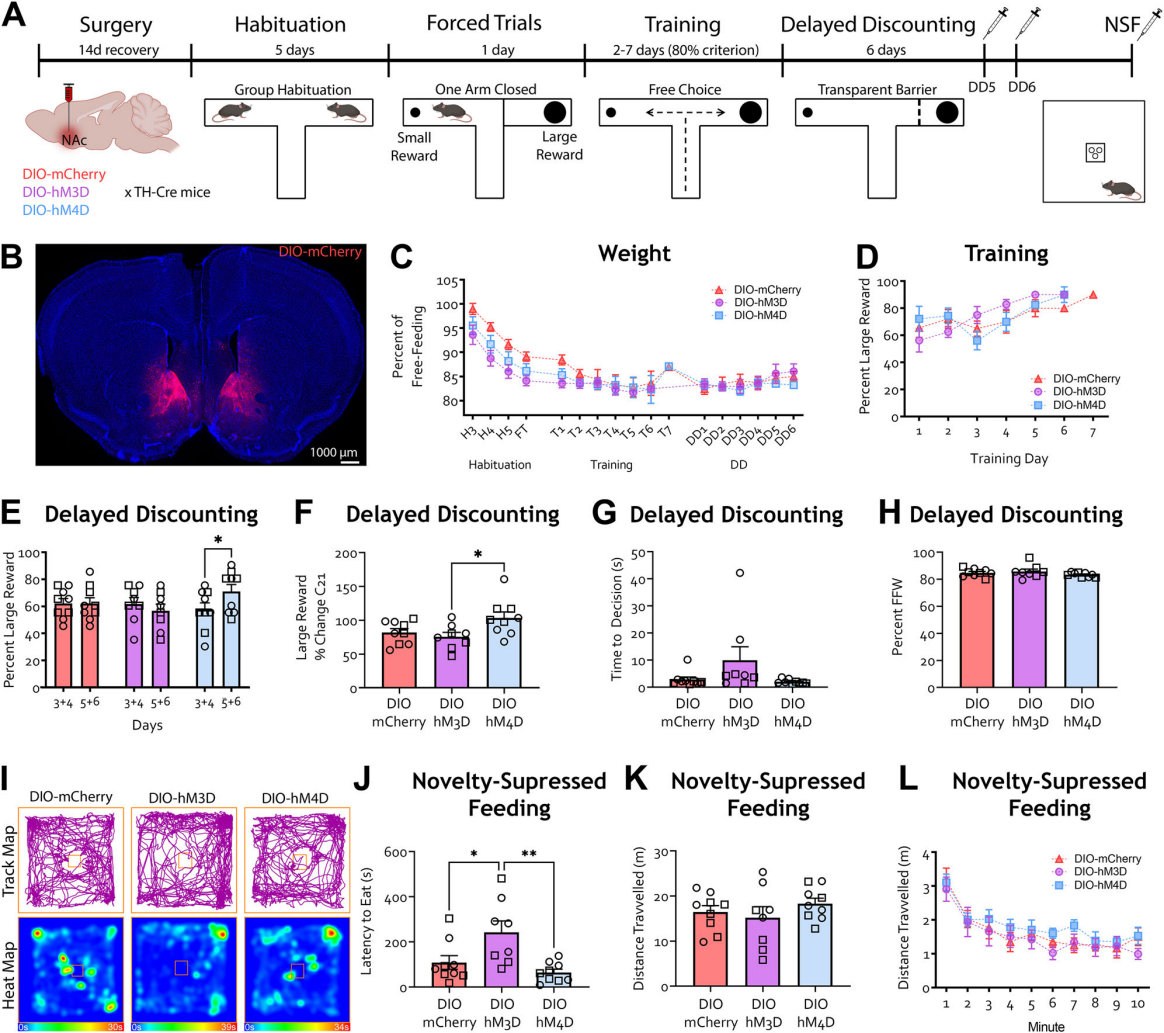
TH-positive NAc neurons modulate impulsive choice. ***A***, Schematic of the experiment. TH-Cre mice were injected with AAV5-DIO-mCherry, AAV5-DIO-hM3D-mCherry, or AAV5-DIO-hM4D-mCherry in the VTA and started habituation to the T-maze 10 d afterwards. Mice were trained to reach a criterion of 80% choice of the large reward to start the delay discounting (DD) phase of the task in which a 10 s delay was introduced upon choosing the large reward. All groups were injected with DREADD agonist C21 1 h prior to testing on DD Days 5 and 6. Three days after DD, animals were injected with C21 1 h prior to undergoing novelty-suppressed feeding (NSF). ***B***, Representative image of viral targeting for the NAc cohort. ***C***, Percent free-feeding weight across the experiment timeline did not differ between groups (mixed-effects analysis, found no main effect of treatment *F*_(2, 23)_ = 1.674, *p* = 0.2095); there was a significant main effect of time only as the animal weight dropped within the first few days following food restriction. ***D***, Percent choice of the large reward did not differ between groups across training days (mixed-effects analysis found no main effect of treatment, *F*_(2, 23)_ = 0.3999, *p* = 0.6750); there was a significant main effect of time only as mice learned the difference between the large and small reward arms during training. ***E***, Percent choice of the large reward was significantly higher during DD Days 5–6 (presence of DREADD agonist C21) compared with DD Days 3–4 (no drug) in hM4D mice. No difference was observed in mCherry or hM3D mice (Bonferroni's multiple-comparisons test, mCherry *p* > 0.9999; hM3d *p* = 0.9015). Additionally, there was no effect of treatment (*F*_(2, 23)_ = 0.3964, *p* = 0.6772) or DD days (*F*_(2, 23)_ = 1.122, *p* = 0.3005). ***F***, Choice of large reward represented as a percent change from the average choices on DD Days 3–4 was significantly higher in hM4D mice compared with hM3D mice (Tukey's multiple-comparisons test, DIO-mCherry vs DIO-hM4D, *p* = 0.1027; DIO-mCherry vs DIO-hM3D, *p* = 0.8200). ***G***, Latency to make a decision on DD Days 5 and 6 did not differ between groups (one-way ANOVA, *F*_(2, 23)_ = 2.552, *p* = 0.0998). ***H***, Percent free feeding weight (FFW) did not differ between groups (one-way ANOVA, *F*_(2, 23)_ = 1.147, *p* = 0.3352). ***I***, Representative track and heat maps of mice during NSF task. ***J***, Latency to eat in the NSF task was higher in hM3D mice compared with mCherry and hM4D mice (Tukey's multiple-comparisons test, DIO-mCherry vs DIO-hM4D, *p* = 0.5906). ***K***, Total distance traveled during the NSF task did not differ between groups (one-way ANOVA, *F*_(2, 23)_ = 0.8363, *p* = 0.4461). ***L***, Distance traveled each minute during NSF task did not differ between groups (two-way rmANOVA, found no main effect of treatment *F*_(2, 23)_ = 0.8363, *p* = 0.4461, or interaction of treatment by time *F*_(18, 207)_ = 0.4282, *p* = 0.9807). *n* = mCherry (9), DIO-hM3D (8), DIO-hM4D (9), *p < 0.05, **p < 0.01. Each data point is represented as a square (male) or a circle (female) for transparency.

For Figures 3 and 5, mixed model analyses were conducted to examine any change in the percent free-feeding weight across the experimental timeline and trajectory of learning performance. One-way ANOVAs were used to compare treatment groups for preference of small reward as a percent change, time to make a decision, percent free-feeding weight, latency to eat, distance traveled in the novelty-suppressed feeding (NSF) task, and c-Fos cell counts. A two-way rmANOVA was used to examine differences in percent choice of small reward between DD Days 3–4 and 5–6 as well as for the minute-by-minute analysis of distance traveled in the NSF task. Significant statistics were reported in the results and nonsignificant statistics in the figure legends.

#### Sex differences

In the analysis of Figure 1 data, we found no significant effects of sex on the number of training days required to meet the learning criterion (two-way ANOVA, no main effect of age group, *F*_(1, 46)_ = 1.53, *p* = 0.2220; sex, *F*_(1, 46)_ = 1.86, *p* = 0.1787; or interaction, *F*_(1, 46)_ = 0.25, *p* = 0.6210) or on the learning curve (three-way repeated measures ANOVA, no interaction, *F*_(8, 368)_ = 0.78, *p* = 0.6187). There were also no sex differences in percent small reward choices at either a 5 s (two-way ANOVA, found a significant main effect of age group, *F*_(1, 21)_ = 26.63, *p* < 0.0001, but no main effect of sex, *F*_(1, 21)_ = 0.62, *p* = 0.4389, or interaction effect of age group by sex, *F*_(1, 21)_ = 0.039, *p* = 0.8447) or 10 s delay (two-way ANOVA, significant main effect of age, *F*_(1, 21)_ = 12.48, *p* = 0.0020, but no main effect of sex, *F*_(1, 21)_ = 2.83, *p* = 0.1074, or interaction, *F*_(1, 21)_ = 0.37, *p* = 0.5476). While no sex differences were observed in the percent entries into the small reward arm across DD days with a 5 s delay (three-way repeated measures ANOVA, no main effect of sex, *F*_(1, 21)_ = 0.031, *p* = 0.8624; no interaction effect of sex by training day, *F*_(5, 105)_ = 1.015, *p* = 0.4129; no interaction effect of sex by training day by age group, *F*_(5, 105)_ = 0.17, *p* = 0.9739), we did find a significant main effect of sex when the delay was 10 s with males entering the small reward arm more than females (three-way repeated measures ANOVA, significant main effect of sex, *F*_(1, 21)_ = 4.96, *p* = 0.0371). This, however, did not coincide with any interaction effects of sex and the delay day, or sex and the delay day and age group (three-way repeated measures ANOVA, *F*_(5, 105)_ = 2.101, *p* = 0.0710, *F*_(5, 105)_ = 1.07, *p* = 0.4005).

In Figure 2, we found no significant effect of sex in the number of days needed to acquire the initial learning criterion (two-way ANOVA, main effect of sex, *F*_(1, 33)_ = 2.41, *p* = 0.1299; main effect of age group, *F*_(1, 33)_ = 4.90, *p* = 0.0339; interaction effect of sex by age group, *F*_(1, 33)_ = 0.21, *p* = 0.6472), but a main effect of sex in the learning curve, with males choosing the large reward arm more than females overall (three-way repeated measures ANOVA, main effect of sex, *F*_(1, 33)_ = 4.37, *p* = 0.0443; interaction effect of training day by sex, *F*_(8, 264)_ = 1.023, *p* = 0.4192; interaction effect of training day by age group by sex, *F*_(8, 264)_ = 0.7483, *p* = 0.6488). No sex differences were found in the number of days needed to meet the learning criterion for the reverse framework (two-way ANOVA, main effect of sex, *F*_(1, 33)_ = 0.19, *p* = 0.6632; main effect of age group, *F*_(1, 33)_ = 1.83, *p* = 0.1850; interaction effect of sex by age group, *F*_(1, 33)_ = 1.47, *p* = 0.2333) and its respective learning curve (three-way rmANOVA, main effect of sex, *F*_(1, 33)_ = 0.88, *p* = 0.3559; interaction effect of sex by training day, *F*_(11, 363)_ = 0.68, *p* = 0.7570; interaction effect of sex by age group by training day, *F*_(11,363) _= 0.5764, *p* = 0.8480).

## Results

A major challenge in developmental research is working within relatively short temporal windows. To assess impulsive choice in mice using a time-constrained experimental design, we first sought to adapt a DD T-maze paradigm more commonly used in rats (Olmstead et al., 2006). The procedure was broken down into four stages (Fig. 1*A*). Animals were first handled and habituated to the T-maze apparatus. Bowls are then placed in each of the two opposing branches of the T-maze, one containing fewer sucrose pellets (i.e., the small reward) and one with more sucrose pellets (i.e., the large reward). Through forced trials, animals equally sampled both reward arms before commencing the choice stage, in which they were free to choose which reward arm to enter. Animals were food restricted and had a natural tendency to choose a larger amount of sucrose pellets. Training days continued until animals displayed a criterion of a stable preference for the large reward arm (>80% preference) for 2 consecutive days. Once a learning criterion was reached, animals underwent 6 d of DD phase, whereby a delay contingency was applied for the large reward, and animals learned that they must wait to retrieve sucrose pellets from the large reward arm.

To measure DD, a task that would span 6 consecutive days, within adolescence [lasting from approximately postnatal day (P)30 to P45 in mice (Spear, 2000b)], we needed to create a learning protocol that would allow mice to distinguish between the two reward arms within a tight timeline from weaning (P21) to midadolescence (∼P35). To do this, we first performed two pilot studies in adult mice, aiming for animals to meet the learning criterion within 9 training days (one session/day), translating to an assessment of DD in the adolescent group from P35 to P41. In our first pilot, only 40% of animals reached the learning criterion after 9 training days, and 11.1% in the second pilot (Extended Data Fig. 1-1*B*). From this, we determined important factors that affect the animals’ abilities to learn the T-maze task. Notably, the amount of handling, group habituation to the apparatus, the difference in magnitude between the small and large rewards, and the number of trials conducted per session (Extended Data Fig. 1-1*C*). In our final protocol, all adult animals met the learning criterion within 9 training days (Extended Data Fig. 1-1*B*).

### Preference for the large reward with a delay contingency is higher in adult mice

To validate this protocol against well-described developmental differences in impulsive choice (Adriani and Laviola, 2003; Pinkston and Lamb, 2011; Doremus-Fitzwater et al., 2012; McClure et al., 2014; Lukkes et al., 2016), we compared the performance of adolescent and adult mice in the T-maze DD task (Fig. 1*A*). All animals exhibited an 80% preference for the large reward arm over 2 consecutive training days with no age-dependent differences in the number of training days required to reach this learning criterion (Fig. 1*B*) or in the learning curve (Fig. 1*C*; two-way rmANOVA only found a significant main effect of training day, *F*_(8, 184)_ = 27.07, *p* < 0.0001).

Next, we assessed the percent choice for the large reward when a 5 s delay contingency was added for the large reward arm in adolescents and adults. This was calculated as the percent entries into the large reward arm averaged across DD Days 5 and 6. Animals in the adolescent group (mean age = 37.17 d, SEM = 0.43) exhibited a significantly lower preference for the larger reward compared with the adult group (mean age = 62.42 d, SEM = 0.35; Fig. 1*D*; unpaired *t* test, *t*_23_ = 5.476, *p* < 0.0001). Age-dependent differences in preference for the large reward were observed during DD Days 4, 5, and 6 (Fig. 1*E*; two-way rmANOVA found a significant main effect of DD day, *F*_(5, 115)_ = 15.35, *p* < 0.0001; significant main effect of age, *F*_(1, 23)_ = 26.83, *p* < 0.0001; significant interaction effect of DD day by age, *F*_(5, 115)_ = 3.941, *p* = 0.0025. Bonferroni's multiple-comparisons test, adolescents vs adults Day 4, *p* = 0.0002; Day 5, *p* < 0.0001; Day 6, *p* < 0.0001).

To test whether increasing the delay would potentiate this effect, we trained a separate group of animals and conducted the DD phase with a 10 s delay. Similar to what we saw with the 5 s delay, adolescent animals in the 10 s delay group (mean age = 36.77, SEM = 0.73) exhibited a significantly lower preference for the large reward compared with their adult counterparts (mean age = 62.14, SEM = 0.34; Fig. 1*G*; unpaired *t* test, *t*_23_ = 3.660, *p* < 0.0013). Age-dependent differences in percent entries into the large reward arm were observed on DD Days 5 and 6 (Fig. 1*H*; two-way rmANOVA found a significant main effect of DD day, *F*_(5, 115)_ = 34.41, *p* < 0.0001; significant main effect of age group, *F*_(1, 23)_ = 6.472, *p* = 0.0181; and significant interaction of DD day by age, *F*_(5, 115)_ = 3.057, *p* = 0.0125. Bonferroni's multiple-comparisons test, adolescent vs adult Day 5, *p* = 0.0042; Day 6, *p* = 0.0056). When comparing the DD curves of adolescent and adult groups between no delay and the 5 s delay, we found an interaction effect of age by delay length (Fig. 1*F*; two-way rmANOVA found a significant main effect of age, *F*_(1, 23)_ = 21.46, *p* = 0.0001; significant main effect of delay length, *F*_(1, 23)_ = 208.9, *p* < 0.0001; significant interaction effect of age by delay length, *F*_(1, 23)_ = 32.48, *p* < 0.0001; Bonferroni's multiple-comparisons test, adolescent vs adult 5 s delay, *p* < 0.0001). The same was observed for DD curve comparisons in the 10 s delay (Fig. 1*I*; two-way rmANOVA found a significant main effect of age, *F*_(1, 23)_ = 13.93, *p* = 0.0011; Bonferroni's multiple-comparisons test, 10 s delay adolescents vs adults, *p* < 0.0001. Significant main effect of delay length, *F*_(1, 23)_ = 236.8, *p* < 0.0001; Bonferroni's multiple-comparisons test, adolescents no delay vs 10 s delay, *p* < 0.0001. Significant interaction effect of age by delay length, *F*_(1, 23)_ = 10.92, *p* < 0.0031). We found no major sex differences in this dataset (see Materials and Methods for details). Differences in percent choice for large reward between adolescent and adult groups were driven neither by differences in percent free-feeding weight at either the 5 s (Extended Data Fig. 1-2*A,B*) or 10 s delay (Extended Data Fig. 1-2*C,D*) nor by differences in their absolute weights (Extended Data Fig. 1-2*E,F*). The equivalent learning rates and lack of correlation between weight and choice suggest that, despite their lower body weight, adolescent mice are still ascribing high relative value to the large reward versus the small reward. Nevertheless, we cannot definitively exclude the possibility that small and large rewards may be closer to the saturation point of adolescent mice compared with adults, thereby reducing the relative value of the large reward.

Two critical assumptions made in this T-maze DD paradigm are that (1) animals have learned that they must wait for a delay to retrieve sucrose from the large reward arm at the time the impulsivity score is measured (i.e., delay Days 5 and 6) and that (2) animals are making a decision, such that their behavior is not driven by a lack of prepotent response inhibition. To test these assumptions, we conducted a reversal learning experiment on a separate naive batch of C57BL/6J animals (Fig. 2*A*), whereby the large and small reward arms were switched the day after the animals reached the learning criterion. Interestingly, adolescent mice in this new cohort met the initial learning criterion approximately 2 d earlier than their adult counterparts (Fig. 2*B*; unpaired *t* test *t*_35_ = 2.218, *p* = 0.0322). Animals effectively learned the difference between large and small reward arms; however, no age-dependent differences were found in the learning curve (Fig. 2*C*; two-way rmANOVA, only found a significant main effect of training day *F*_(8, 280)_ = 15.45, *p* < 0.0001). Once the animals met the learning criterion, the large and small reward arms were swapped, such that the original large reward arm now contained only one sucrose pellet, and the original small reward arm contained six sucrose pellets. The adolescent and adult groups learned this new reversed framework at a similar rate (Fig. 2*D,E*; learning curve, two-way rmANOVA, only found a significant main effect of reversal learning day, *F*_(11, 385)_ = 71.72, *p* < 0.0001), suggesting that age-dependent differences in behavioral flexibility did not drive the heightened DD observed in adolescent mice. Similarly to before, we found no significant sex differences in this dataset (see Materials and Methods for details).

### Chemogenetic inhibition of NAc TH-positive neurons leads to a higher preference for the large reward with a delay contingency

In a separate set of experiments, we next used this T-maze DD task to explore the contributions of mesolimbic neural activity to impulsive choice behavior. Given that the contribution of NAc MSN subtypes and NAc dopamine signaling in DD (Winstanley et al., 2005; Saddoris et al., 2015; Orsini et al., 2017) is unclear, we first decided to bidirectionally modulate the activity of TH-positive neurons in the NAc during DD. We injected the NAc of TH-Cre mice with adeno-associated virus (AAV) expressing Cre-dependent excitatory (DIO-hM3D) or inhibitory (DIO-hM4D) DREADDs or a control vector (DIO-mCherry; Fig. 3*A,B*; Extended Data Fig. 3-1), mostly concentrated on the NAc core and dorsomedial shell. We confirmed that the location of DREADD-expressing NAc MSNs corresponded to a subpopulation of tdTomato-positive NAc MSNs in TH-Cre/Ai9 mice (Extended Data Figs. 3-1*A,B*, 3-2). Following recovery from surgery and completing the training phase, we injected the DREADD agonist C21 1 h prior to testing on DD Days 5 and 6 (Jendryka et al., 2019; Botterill et al., 2024; Fig. 3*A*). We saw no difference in free-feeding weight between treatment groups (Fig. 3*C*; mixed-effects analysis only found a significant main effect of time *F*_(16, 316)_ = 39.18, *p* < 0.0001) as mice lost weight in the first few days following food restriction. Performance during training did not differ between treatment groups (Fig. 3*D*; mixed-effects analysis only found a significant main effect of training day *F*_(6, 75)_ = 7.589, *p* < 0.0001) as mice learned the difference between small and large rewards during training. During DD, inhibition of NAc TH-positive neurons (hM4d) led to an increase in the percent choice of the large reward, with no effect from activation (Fig. 3*E*; two-way rmANOVA main effect of interaction *F*_(2, 23)_ = 4.358, *p* = 0.0248; Bonferroni's multiple-comparisons test, DIO-hM4d *p* = 0.0194). Inhibition of NAc TH-positive neurons led to a higher choice of large reward represented as an average percent change from DD Days 3–4 to DD Days 5–6 compared with activation (Fig. 3*F*; one-way ANOVA *F*_(2, 23)_ =4.067, *p* = 0.0307, Tukey's post hoc tests DIO-hM3D vs DIO-hM4D *p* = 0.0341), suggesting decreased impulsive choice upon inhibition of TH-positive NAc neurons. When we looked at latency to make a decision during DD Days 5 and 6, we found no difference between treatment groups (Fig. 3*G*). Nevertheless, future experiments need to directly address a possible effect on reward magnitude with a no-delay contingency. Percent free-feeding weight did not differ between treatments (Fig. 3*H*).

10.1523/ENEURO.0487-24.2024.f3-1 Figure 3-1 **Viral targeting maps for NAc surgeries. A.** Representative images of viral (mCherry control) targeting in coronal sections spanning the NAc of TH-Cre mice. **B-D.** Representative maps of overall viral expression in coronal sections spanning the NAc of mCherry control (**B**), hM3d (**C**) and hM4d (**D**) injected mice. Download Figure 3-1, TIF file.

10.1523/ENEURO.0487-24.2024.f3-2 Figure 3-2 **Characterization of NAc TH positive population. A.** Representative images of td-Tomato positive cells in coronal sections spanning the NAc in TH-Cre x ai9 mice **B.** Representative images of viral targeting in coronal sections spanning the NAc in TH-Cre mice **C.** Representative diagrams of overall td-Tomato expression in coronal sections spanning the NAc of TH-Cre x ai9 mice. Download Figure 3-2, TIF file.

To functionally validate our DREADD manipulation of TH-positive neurons in the NAc, mice underwent novelty-suppressed feeding (NSF) approximately 5 d after completing DD. We chose NSF because of work previously showing this task is modulated by NAc manipulations (Burghardt et al., 2016; Cheng et al., 2018). Activation of TH-positive NAc neurons increased latency to feed compared with control and hM4D animals (Fig. 3*I,J*; one-way ANOVA *F*_(2, 24)_ = 7.176, *p* = 0.0038, Tukey's multiple-comparisons post hoc test DIO-hM3D vs DIO-hM4D *p* = 0.0036 and DIO-hM3D vs DIO-mCherry *p* = 0.0291). Importantly, while we did not see an effect of NAc TH-positive neuron inhibition on latency to eat in the NSF task, NAc activity has been implicated in the modulation of anxiety-like behavior (Barrot et al., 2002; Kim et al., 2008; Bahi and Dreyer, 2019), which could have contributed to our effects. Additionally, distance traveled during the NSF task did not differ across treatments (Fig. 3*K,L*; minute by minute, two-way ANOVA main effect of time only *F*_(9, 25)_ = 20.54, *p* < 0.0001).

**Figure 4.**
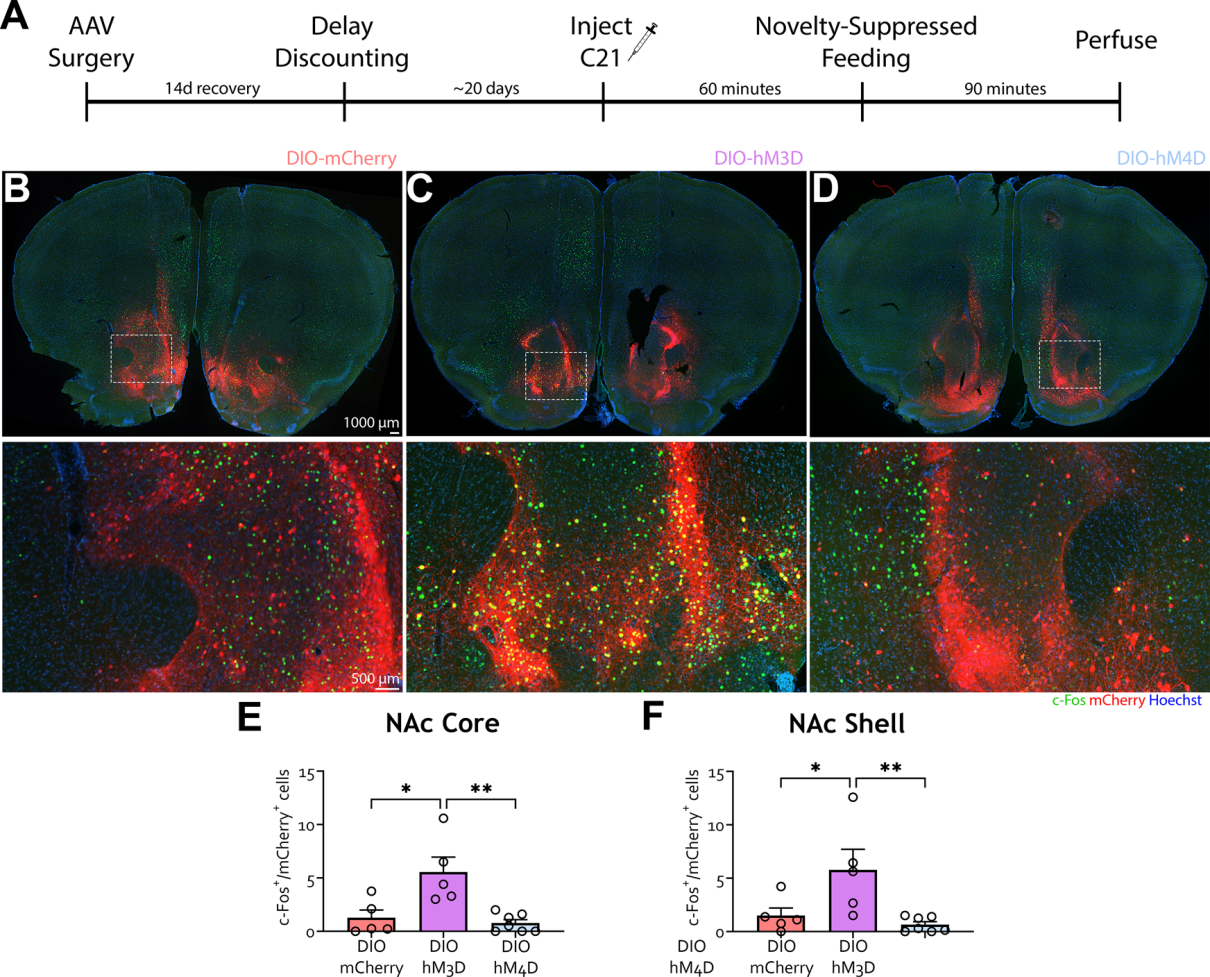
c-Fos validation of NAc chemogenetic manipulations. ***A***, Schematic of the experiment. TH-Cre mice were injected with AAV5-DIO-mCherry, AAV5-DIO-hM3D-mCherry, or AAV5-DIO-hM4D-mCherry in the NAc and started habituation to the T-maze 10 d afterward. All groups were injected with DREADD agonist C21 1 h prior to testing on DD Days 5 and 6. Three days after DD, animals were injected with C21 1 h prior to undergoing novelty-suppressed feeding (NSF) and were perfused 90 min later for brain processing for c-Fos immunohistochemistry. ***B–D***, Representative images showing viral (mCherry) and c-Fos (green) expression with nuclear dye Hoechst (blue) in the NAc of mice injected with mCherry (***B***), hM3D (***C***), and hM4D (***D***). ***E***, Number of c-Fos–positive cells with viral expression in the NAc core was significantly higher in hM3D mice compared with mCherry and hM4D (Tukey's multiple-comparisons tests, DIO-mCherry vs DIO-hM4D, *p* = 0.9037). ***F***, Number of c-Fos–positive cells with viral expression in the NAc shell was significantly higher in hM3D mice compared with mCherry and hM4D (Tukey's multiple-comparisons tests, DIO-mCherry vs DIO-hM4D, *p* = 0.8378). *n* = mCherry (5), DIO-hM3D (5), DIO-hM4D (7), *p < 0.05, **p < 0.01.

At 90 min following the NSF task, animals were euthanized, and brains were harvested to evaluate the expression of the immediate early gene c-Fos with C21 treatment (Fig. 4*A–D*). Activation of NAc MSNs led to an increase in c-Fos expression in the NAc core (Fig. 4*E*; one-way ANOVA, *F*_(2, 14)_ = 9.647, *p* = 0.0023, Tukey's multiple-comparisons post hoc tests, DIO-mCherry vs DIO-hM3D, *p* = 0.0103; DIO-hM3D vs DIO-hM4D, *p* = 0.0026) and shell (Fig. 4*F*; one-way ANOVA, *F*_(2, 14)_ = 6.521, *p* = 0.0100, Tukey's multiple-comparisons post hoc tests, DIO-mCherry vs DIO-hM3D, *p* = 0.0434; DIO-hM3D vs DIO-hM4D, *p* = 0.0096). Together, these data suggest that TH-positive neurons in the NAc modulate impulsive choice during DD.

### Chemogenetic manipulation of TH-positive VTA neurons has no effect on impulsive choice

**Figure 5.**
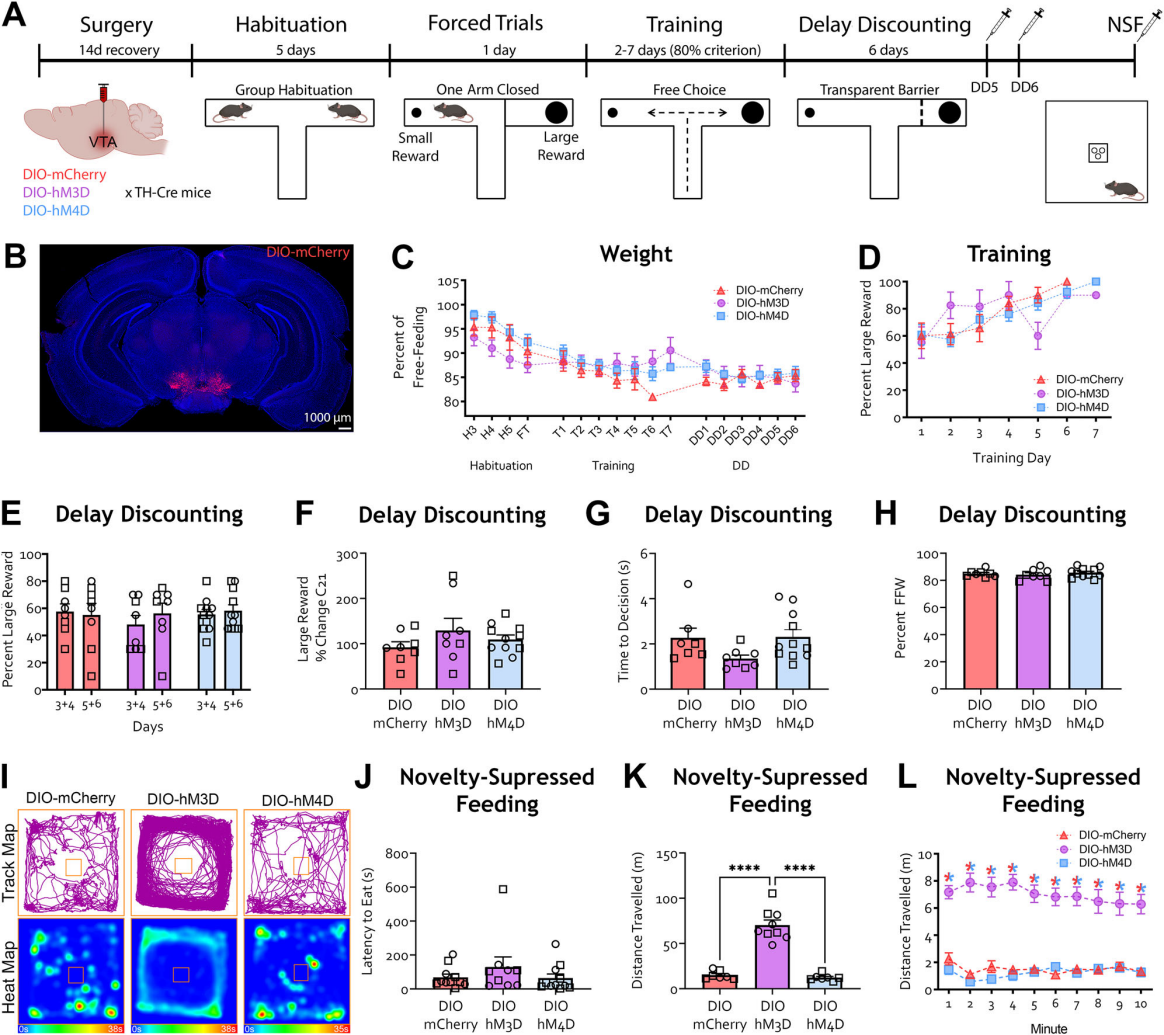
Inhibition of VTA TH-positive neurons does not alter impulsive choice. ***A***, Schematic of experiment. TH-Cre mice were injected with AAV5-DIO-mCherry, AAV5-DIO-hM3D-mCherry, or AAV5-DIO-hM4D-mCherry in the VTA and started habituation to the T-maze 10 d afterward. Mice were trained to reach a criterion of 80% choice of the large reward to start the delay discounting (DD) phase of the task in which a 10 s delay was introduced upon choosing the large reward. All groups were injected with DREADD agonist C21 1 h prior to testing on DD Days 5 and 6. Three days after DD, animals were injected with C21 1 h prior to undergoing novelty-suppressed feeding (NSF). ***B***, Representative image of viral targeting for the VTA cohort. ***C***, Weight distributions across the experimental timeline did not differ between groups (mixed-effects analysis found no main effect of treatment *F*_(2, 24)_ = 0.8621, *p* = 0.4349). There was a significant main effect of time only as the animal weight dropped within the first few days following food restriction. ***D***, Percent choice of the large reward did not differ between groups during training (mixed-effects analysis found no main effect of treatment, *F*_(2, 24)_ = 1.095, *p* = 0.3506); there was however a main effect of time only as mice learned the difference between the large and small reward arms. ***E***, Percent choice of the large reward during DD days did not differ between DD Days 3–4 (with the absence of C21) and DD Days 5–6 (presence of DREADD agonist C21) for any of the groups (two-way ANOVA found no main effect of treatment *F*_(2, 24)_ = 0.2574, *p* = 0.7752; time *F*_(1, 24)_ = 0.5161, *p* = 0.4794; or treatment–time interaction *F*_(2, 24)_ = 0.5696, *p* = 0.5732). ***F***, Choice of large reward represented as a percent change from the average choices on DD Days 3–4 did not differ between groups (one-way ANOVA, *F*_(2, 24)_ = 1.123, *p* = 0.3419). ***G***, Latency to make a decision on DD Days 5 and 6 did not differ between groups (one-way ANOVA, *F*_(2, 22)_ = 2.762, *p* = 0.0851). ***H***, Percent FFW did not differ between groups (one-way ANOVA, *F*_(2, 24)_ = 0.3800, *p* = 0.6879). ***I***, Representative track and heat maps of mice during NSF task. ***J***, Latency to feed in the NSF task did not differ between groups (one-way ANOVA, *F*_(2, 28)_ = 1.010, *p* = 0.3771). ***K***, Total distance traveled during the NSF task was significantly higher in hM3D mice compared with mCherry and hM4D mice (Bonferroni's multiple-comparisons test, DIO-mCherry vs DIO-hM4D, *p* = 0.9009). ***L***, Distance traveled each minute during the NSF task was higher in hM3D mice compared with mCherry and hM4D mice (Bonferroni's multiple-comparisons test, DIO-mCherry vs DIO-hM4D, *p* > 0.9999). There was no main effect of time (*F*_(9, 180)_ = 0.8545, *p* = 0.5671). *n* = mCherry (8), DIO-hM3D (8), DIO-hM4D (11), *p < 0.05, ** *p* < 0.01. Each data point is represented as a square (male) or a circle (female) for transparency.

Dopaminergic innervation of the NAc predominantly stems from the VTA (Phillipson and Griffiths, 1985; S. Ikemoto, 2007; Morales and Margolis, 2017). We next tested if changes in dopaminergic cell activity in the VTA might similarly affect impulsive behavior in this task. We injected the VTA of TH-Cre mice with AAV virus expressing Cre-dependent excitatory (DIO-hM3D), inhibitory (DIO-hM4D) DREADD, or control (DIO-mCherry) virus (Fig. 5*A,B*; Extended Data Fig. 5-1). Animals across treatment groups showed similar weight across the experiment timeline (Fig. 5*C*; mixed-effects analysis only found a significant main effect of time, *F*_(16, 345)_ = 30.48, *p* < 0.0001) and training performance (Fig. 5*D*, mixed-effects analysis only found a significant main effect of time *F*_(6, 84)_ = 9.601, *p* < 0.0001). Preference for the large reward was not affected by inhibition or activation of VTA TH-positive neurons (Fig. 5*E*) nor was the percent change (Fig. 5*F*). Likewise, latency to make a decision (Fig. 5*G*) and percent free-feeding weight (Fig. 5*H*) were similar between groups. Moreover, we saw no difference in latency to eat during the NSF task (Fig. 5*J*). However, activation of VTA TH-positive neurons in DIO-hM3D–injected mice led to an increase in distance traveled compared with hM4D and mCherry control animals (Fig. 5*K*; one-way ANOVA, *F*_(2, 20)_ = 67.75, *p* < 0.0001; Tukey's multiple-comparisons post hoc test, DIO-mCherry vs DIO-hM3D, *p* < 0.0001; DIO-hM4D vs DIO-hM3D, *p* < 0.0001). This was also reflected in the minute-by-minute analysis of distance traveled (Fig. 5*L*; two-way ANOVA found a significant main effect of treatment, *F*_(2, 20)_ = 67.75, *p* < 0.0001; Bonferroni's multiple-comparisons test, DIO-mCherry vs DIO-hM3D, *p* < 0.0001; DIO-hM4D vs DIO-hM3D, *p* < 0.0001; a significant main effect of treatment by time interaction, *F*_(18,180)_ = 2.197, *p* = 0.0048). The enhancement of locomotor effects following VTA dopaminergic activation is consistent with previous evidence (Boekhoudt et al., 2016; Jing et al., 2019).

10.1523/ENEURO.0487-24.2024.f5-1 Figure 5-1 **Viral targeting maps for VTA surgeries. A.** Representative images of viral expression in mice injected with a control (mCherry) virus. Coronal sections spanning the VTA depict viral expression as mCherry (red) and nuclear dye Hoechst (blue). **B-D** Representative diagrams of overall viral expression in mice injected with mCherry (**B**) hM3d (**C**) and hM4d (**D**). Download Figure 5-1, TIF file.

**Figure 6.**
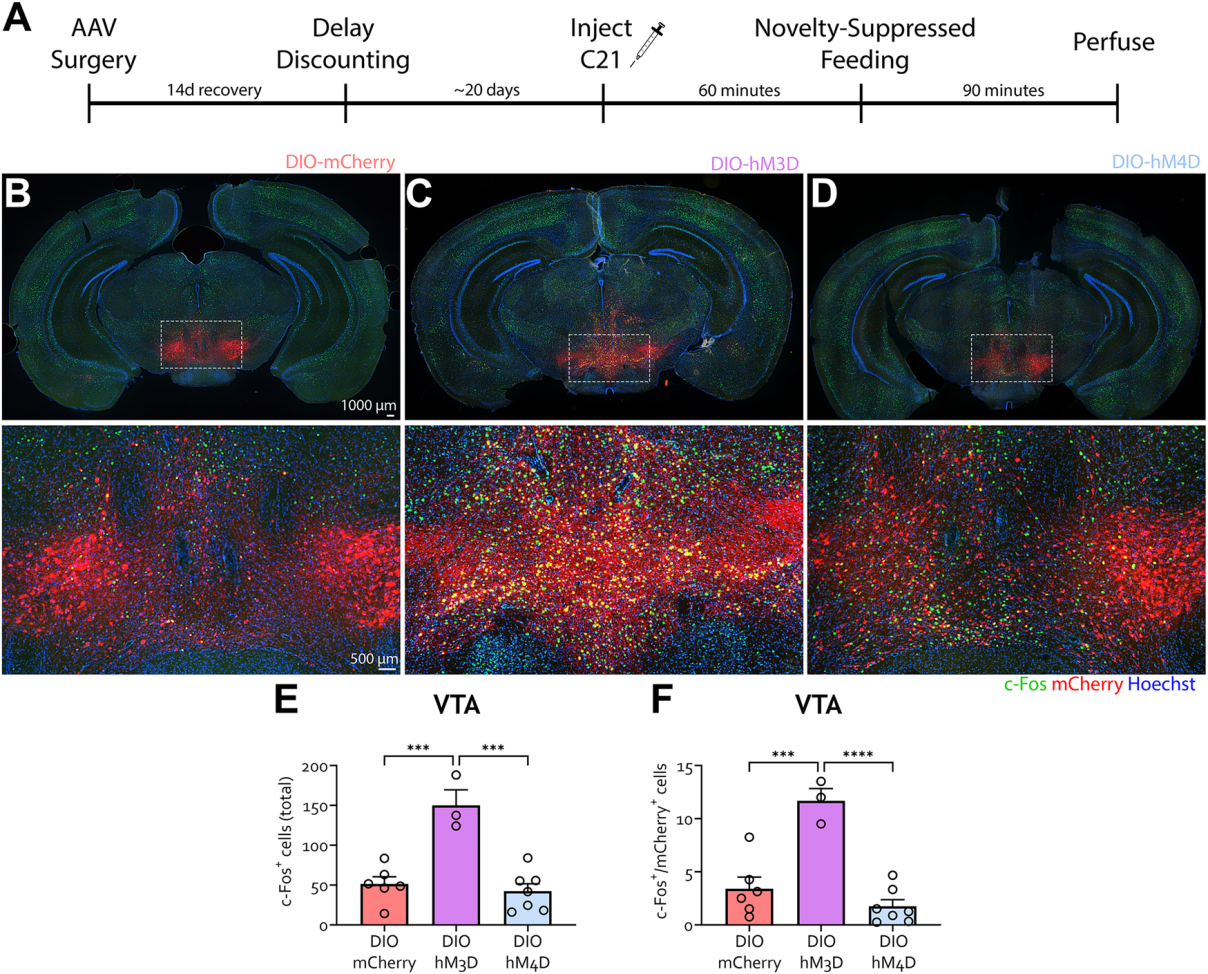
c-Fos validation of VTA chemogenetic manipulations. ***A***, Schematic of the experiment. TH-Cre mice were injected with AAV5-DIO-mCherry, AAV5-DIO-hM3D-mCherry, or AAV5-DIO-hM4D-mCherry in the VTA and started habituation to the T-maze 10 d afterward. All groups were injected with DREADD agonist C21 1 h prior to testing on DD Days 5 and 6. Three days after DD, animals were injected with C21 1 h prior to undergoing novelty-suppressed feeding (NSF). Animals were euthanized 90 min later, and following perfusion, their brains were harvested and processed for imaging. ***B–D***, Representative images showing viral (mCherry) and c-Fos (green) expression with nuclear dye Hoechst (blue) in the VTA of mice injected with mCherry (***B***), hM3D (***C***), and hM4D (***D***). ***E***, Total number of c-Fos–positive cells in the VTA was higher in hM3D mice compared with mCherry and hM4D (Tukey's multiple-comparisons test, mCherry vs hM4d, *p* = 0.8082). ***F***, Number of c-Fos–positive cells with viral expression in the VTA was higher in hM3D mice compared with mCherry and hM4D (Tukey's multiple-comparisons test, mCherry vs hM4D, *p* = 0.3798). *n* = mCherry (6), DIO-hM3D (3), DIO-hM4D (7), *p < 0.05, **p < 0.01.

Animals were euthanized 90 min following the NSF task, and brains were harvested to evaluate the c-Fos expression with the C21 treatment (Fig. 6*A,D*). VTA TH activation (DIO-hM3D) led to a significantly higher number of c-Fos–positive cells compared with DIO-mCherry and DIO-hM4D mice (Fig. 6*E*; one-way ANOVA, *F*_(2, 13)_ = 20.15, *p* = 0.0001; Tukey's multiple-comparisons test, DIO-mCherry vs DIO-hM3D, *p* = 0.0003; DIO-hM3D vs DIO-hM4D, *p* = 0.0001). Likewise, cells with viral and c-Fos expression were higher in hM3D mice compared with mCherry and hM4D (Fig. 6*F*; one-way ANOVA, *F*_(2, 13)_ = 23.01, *p* < 0.0001; Tukey's multiple-comparisons test, DIO-mCherry vs DIO-hM3D, *p* = 0.0003; DIO-hM3D vs DIO-hM4D, *p* < 0.0001). Together, these data indicate that somatic modulation of VTA TH-positive neurons does not affect DD in this T-maze task.

## Discussion

Here, we used a T-maze DD task to interrogate the neural requirements of impulsive choice in mice. We found that modulation of TH-positive NAc MSNs alters impulsive choice in a T-maze DD task, with inhibition of this cell population driving lower impulsivity. Inhibition of TH-positive neurons in the VTA did not affect decision-making during DD. The relatively short length of training in our task also allowed us to probe developmental changes in impulsive choice. As seen with other tasks capturing developmental modulation of novelty-seeking and risk-taking (Adriani and Laviola, 2003; Pinkston and Lamb, 2011; Doremus-Fitzwater et al., 2012; McClure et al., 2014; Lukkes et al., 2016), we found an increase in impulsive choice in adolescent C57BL/6J mice compared with adults. Together, our data introduce an additional tool for exploring the neural basis of DD in mice across the lifespan and point to cell-specific contributions to impulsive choice within the NAc microcircuit with important implications for neurodevelopment.

Adolescent mice showed an increased preference for the immediate small reward over waiting for the delayed large reward compared with adults regardless of the length of the delay, consistent with previous findings from Sprague Dawley rats (Doremus-Fitzwater et al., 2012; McClure et al., 2014; Lukkes et al., 2016). Research in adolescent impulsivity in mice has predominantly focused on impulsive action (Ciampoli et al., 2017; Remmelink et al., 2017; Sasamori et al., 2018; Afshar et al., 2020; Bouchatta et al., 2020), but DD studies using an operant task have reported increased impulsive choice in adolescent C57BL/6J and CD1 (Adriani and Laviola, 2003; Pinkston and Lamb, 2011), but not DBA/2J (Pinkston and Lamb, 2011) mice. Using a novel automated open-source homecage design, Lee et al. also observed increased impulsive action in adolescent C57BL6/129J mice (J. H. Lee et al., 2020). Interestingly, while in our task adolescent mice behaved equivalently at both the 5 and 10 s delay intervals, adult mice displayed more impulsive choices at the 10 s delay interval. This suggests that this T-maze task may be able to capture age-dependent differences in discounting rates, consistent with other studies (Adriani and Laviola, 2003; Pinkston and Lamb, 2011). We saw no developmental differences in reversal learning between age groups. This is in contrast to studies showing that juvenile mice exhibit less perseverant behaviors on multiple choice reversal learning than adult counterparts (Johnson and Wilbrecht, 2011). Nevertheless, the equivalent reversal learning in adolescent and adult mice on the T-maze suggests that the differences in impulsive choice were not driven by age-dependent alterations in cognitive and behavioral flexibility.

In the second part of this study, we found that inhibition of a select neuronal subpopulation in the NAc core and dorsomedial shell decreased impulsive choice. These findings contrast with several studies showing that NAc core lesions increase impulsive choice (Cardinal et al., 2001; Pothuizen et al., 2005; Bezzina et al., 2007; da Costa Araújo et al., 2009; Valencia-Torres et al., 2012). Nonetheless, a study by Steele and colleagues suggests these NAc core lesion effects may be driven at least in part by deficits in delay preferences and reward magnitude sensitivity (Steele et al., 2018). Furthermore, partial pharmacological inactivation of the NAc core decreases impulsive choice (Moschak and Mitchell, 2014), suggesting that compensation may also contribute to the results seen in lesion studies. Finally, combined NAc core and shell lesions also decrease impulsive choice in delay discounting tasks (Acheson et al., 2006; Koloski et al., 2024). Given that our viral infusions mostly targeted TH-positive NAc neurons in both the core and dorsomedial shell, future work must clarify whether there are subregional differences in the contribution of TH-positive NAc neurons to impulsive choice and whether they extend to other NAc neuronal subtypes.

While a considerable volume of literature points to specialized contributions of NAc MSN subpopulations to appetitively and aversively motivated behavior (Soares-Cunha et al., 2016, 2020; Francis and Lobo, 2017; Castro and Bruchas, 2019; G. Chen et al., 2023), to our knowledge, this is the first study to assign a functional role for TH-positive neurons in the NAc. TH-positive neurons have been identified in the NAc (Björklund and Dunnett, 2007; Ugrumov, 2009) in humans (K. Ikemoto et al., 1998; Cossette et al., 2005a), monkeys (K. Ikemoto et al., 1996, 1998), rats (Voorn et al., 1986; Tashiro et al., 1990; Mura et al., 1995), and mice (Bupesh et al., 2014; Depboylu, 2014; Depboylu et al., 2014; R. Chen et al., 2021). Consistent with previous reports (Voorn et al., 1986; Tashiro et al., 1990), the NAc TH-positive cell population targeted in our manipulation had a round or oval cell body, which ranged from 10 to 20 um in diameter. As previously reported (Depboylu et al., 2014), the increased copy number of the transgene in bacterial artificial chromosome transgenic lines and the bypassing of TH transcriptional regulation (Gong et al., 2003) likely account for the larger population of NAc Ai9 (and DREADD) positive cells compared with that labeled by TH-staining alone. TH-positive neurons are typically linked to dopamine (DA) synthesis (Daubner et al., 2011), and NAc DA signaling has previously been associated with DD decision-making (Saddoris et al., 2015; Orsini et al., 2017), but not in impulsive choice per se. For instance, intra-NAc amphetamine treatment in rats has been shown to increase impulsive choice when the delay is progressively decreased within session and to decrease impulsive choice when the delay is increased (Orsini et al., 2017), indicating that DA may instead contribute to modulating adaptive choice strategies. In another study, 6OHDA-induced intra-NAc DA depletion (70–75%) failed to elicit any deficits in DD (with an ascending increase in delay) and only transiently potentiated the ability of amphetamine to decrease impulsive choice (Winstanley et al., 2005). These results are seemingly in contrast with our findings, wherein TH-positive neurons were found to be critical in driving impulsive choice behavior. However, it is of note that impulsive choice was assessed only at one delay (10 s) in our study.

Importantly, TH-positive neurons in the striatum frequently lack expression of other enzymes required for dopaminergic synthesis and secretion (e.g., aromatic amino acid decarboxylase or vesicular monoamine transporter 2; Weihe et al., 2006; Björklund and Dunnett, 2007; Ugrumov, 2009), questioning whether NAc TH-positive neurons invariably synthesize and release dopamine. Interestingly, striatal TH-positive neurons display morphological, neurochemical, and electrophysiological features of interneurons (Cossette et al., 2005b; Björklund and Dunnett, 2007; Tepper et al., 2010; Ünal et al., 2011; Ibáñez-Sandoval et al., 2015), suggesting the same might be true of the NAc TH-positive population. Indeed, a single-cell census of NAc neuronal subtypes conducted using scRNA-seq and FISH classified NAc TH-positive neurons as interneurons, subdivided into subtypes expressing thyrotropin-releasing hormone receptor (*Trh*) and calbindin 2 (*Calb2*; R. Chen et al., 2021), in line with previously described heterogeneity within TH-positive interneurons in the striatum (Tepper et al., 2010; Ibáñez-Sandoval et al., 2015). Cholinergic D2–expressing interneurons in the NAc were recently implicated in impulsive choice (Cavallaro et al., 2023) but are seemingly distinct from the NAc TH-positive population (R. Chen et al., 2021). This points to highly specialized regulation of impulsive choice by NAc interneuron subtypes. Nevertheless, while this further validates and expands the characterization of this NAc TH-positive subpopulation, its precise connectivity, neurochemistry, and electrophysiological profile require further investigation.

Unexpectedly, we did not see an effect of VTA dopaminergic stimulation on impulsive choice. VTA cue-evoked firing is modulated by delay (Roesch et al., 2007), with alterations in dopaminergic signaling in the VTA affecting impulsive choice (Bernosky-Smith et al., 2018). Importantly, VTA dopamine release in the NAc correlates with DD (Day et al., 2010; Saddoris et al., 2015), and stimulation of this pathway modulates DD decision-making (Saddoris et al., 2015). Furthermore, somatic optogenetic (Flores-Dourojeanni et al., 2021) or chemogenetic (Boekhoudt et al., 2017) stimulation of VTA TH-positive neurons does not affect impulsive choice in a five-choice serial reaction test, but stimulation of VTA-NAc shell increases impulsivity under longer delays (Flores-Dourojeanni et al., 2021), suggesting a dissociation between somatic and pathway-specific influences which might have contributed to our negative results, and that should be further interrogated to resolve the contribution of NAc dopamine to impulsive choice.

Overall, these data show that this T-maze DD task is effective at measuring impulsive choice in C57BL/6J mice, successfully capturing heightened DD in adolescence, and highlight a unique contribution of TH-positive NAc neurons to impulsive choice, thereby expanding our understanding of the contribution of select NAc cell populations to DD.

## Synthesis

Reviewing Editor: Mark Laubach, American University Decisions are customarily a result of the Reviewing Editor and the peer reviewers coming together and discussing their recommendations until a consensus is reached. When revisions are invited, a fact-based synthesis statement explaining their decision and outlining what is needed to prepare a revision will be listed below. The following reviewer(s) agreed to reveal their identity: NONE. Note: If this manuscript was transferred from JNeurosci and a decision was made to accept the manuscript without peer review, a brief statement to this effect will instead be what is listed below.

In their revisions, the authors, as far as I could tell, addressed all concerns raised in the reviews at the Journal of Neuroscience. The study seems solid and reports novel findings.
