# Supplementary figures and images for: Tyrosine Hydroxylase–Positive Nucleus Accumbens Neurons Influence Delay Discounting in a Mouse T-Maze Task
Source: eNeuro. 2024 Dec 12;11(12):ENEURO.0487-24.2024. doi: 10.1523/ENEURO.0487-24.2024 (PMC13172978; doi:10.1523/ENEURO.0487-24.2024)

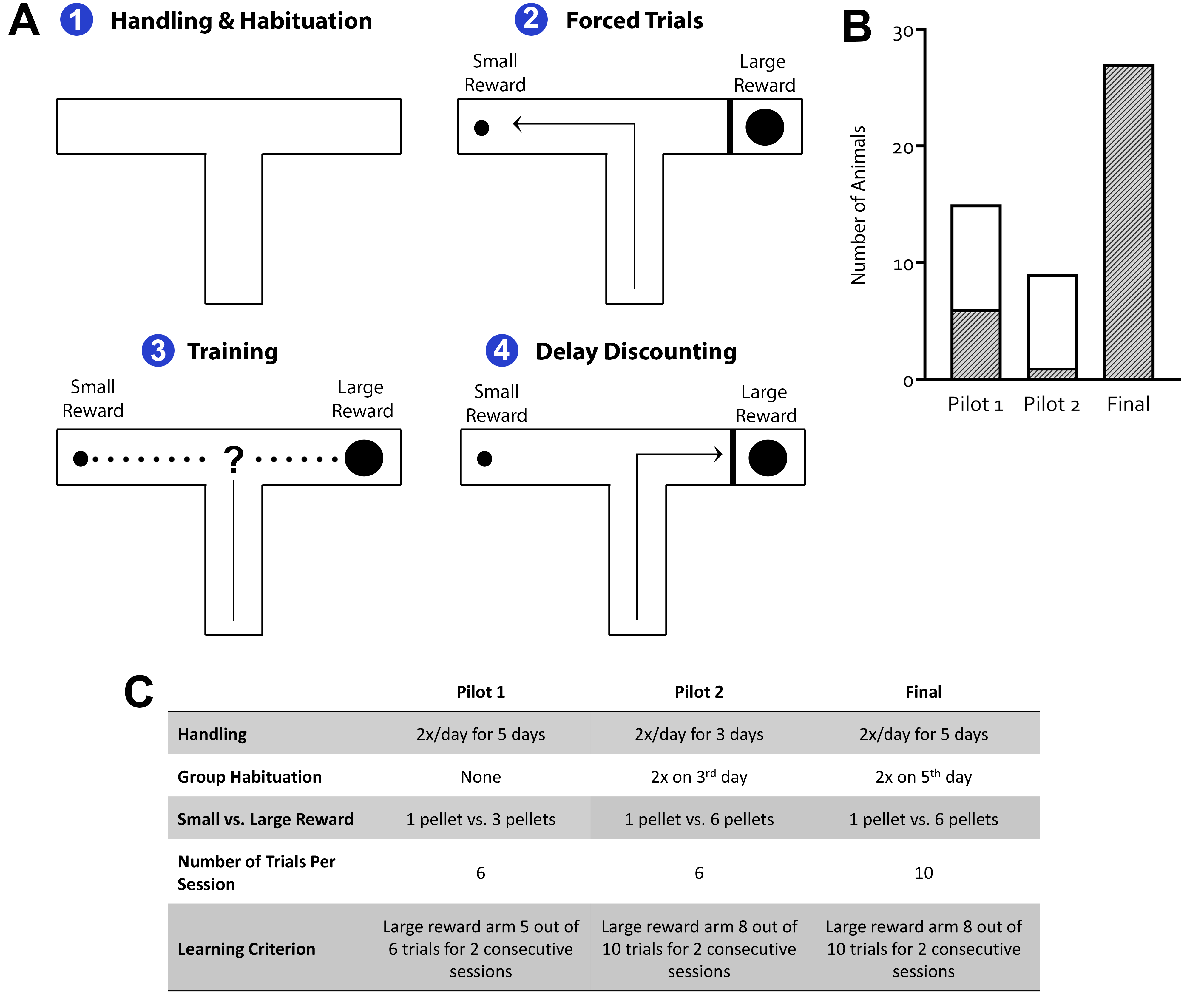

Supplement: Figure 1-1 — Optimization of the T maze DD task. A. Schematic of experiment. The T-maze is comprised of three arms: the starting arm and two reward arms, containing a different amount of sucrose pellets. The task is broken down into four stages: handling and habituation, forced trials, training, and delay discounting (DD). Food restricted animals underwent a training phase and learned the difference between a large and small reward arm (counterbalanced between the left and right T-maze arms across mice). Training phase ended when animals reached criterion of a preference for the large reward in 8 out of 10 trials across 2 consecutive days. Next, in the DD phase (6 days), a 5 or 10 second delay contingency was added for the large reward arm. B. Quantification of the number of animals that reached the learning criterion (grey) or failed to complete training (white). Pilot 1 had 40% of animals reach learning criterion, Pilot 2 had 11.1% and the final DD paradigm had 100% C. Table outlining parameters used for each pilot and the final optimized DD paradigm. Download Figure 1-1, TIF file. [file eneuro-11-ENEURO.0487-24.2024-s001.tif]

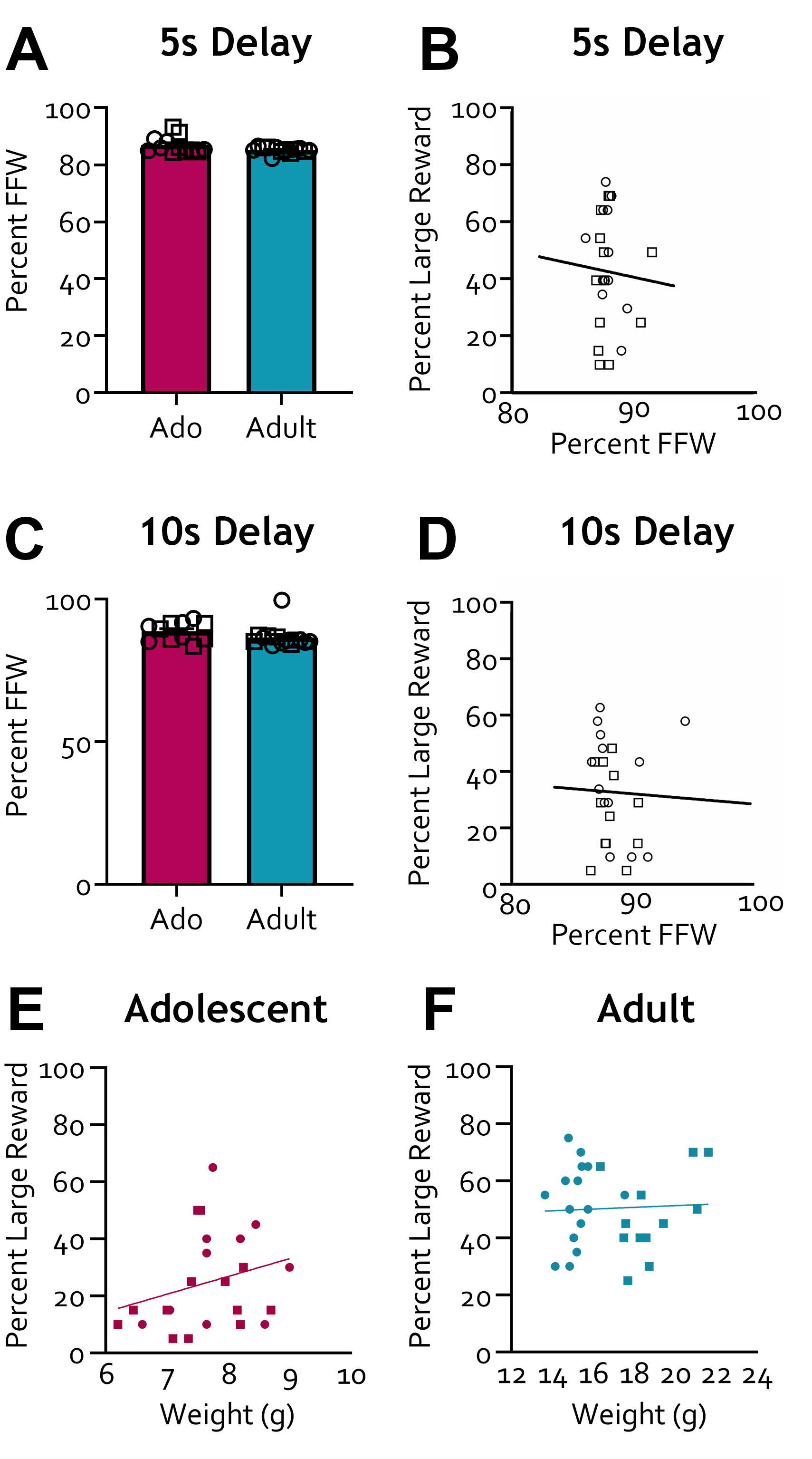

Supplement: Figure 1-2 — Relationship between percent free-feeding weight and choice of small reward in C57BLK6/J adolescent and adult mice. A. Percent free-feeding weight averaged across delay days 5 and 6 did not differ between the adolescent and adult groups (t23 = 2.001, p = 0.0574). B. Simple linear regression of an animal’s percent free-feeding weight averaged across delay days 5 and 6 and the percent choice of large reward with a 5 s delay contingency (ß = -0.9312, F1, 23 = 0.2662, p = 0.6108, R2 = 0.01144). C. Percent free-feeding weight averaged across delay days 5 and 6 did not differ between the adolescent and adult groups (t23 = 1.440, p = 0.1634). D. Simple linear regression of an animal’s percent free-feeding weight averaged across delay days 5 and 6 and the percent choice of large reward with a 10 s delay contingency (ß = -0.3644, F1, 23 = 0.1229, p = 0.7291, R2 = 0.005314). Adolescents, n = 12 (5 females, 7 males); adults, n = 13 (7 females, 6 males). E-F. Simple linear regression of animal weight averaged across delay days 5 and 6 and the percent choice of large reward for adolescent (E: p = 0.2145, R2 = 0.07237) and adult (F: p = 0.8173, R2 = 0.002175) mice. Adolescents, n = 15 (7 females, 8 males); adults, n = 17 (8 females, 9 males). *p < 0.05, ** p < 0.01. Each data point is represented as a square (male) or a circle (female) for transparency. Download Figure 1-2, TIF file. [file eneuro-11-ENEURO.0487-24.2024-s002.tif]

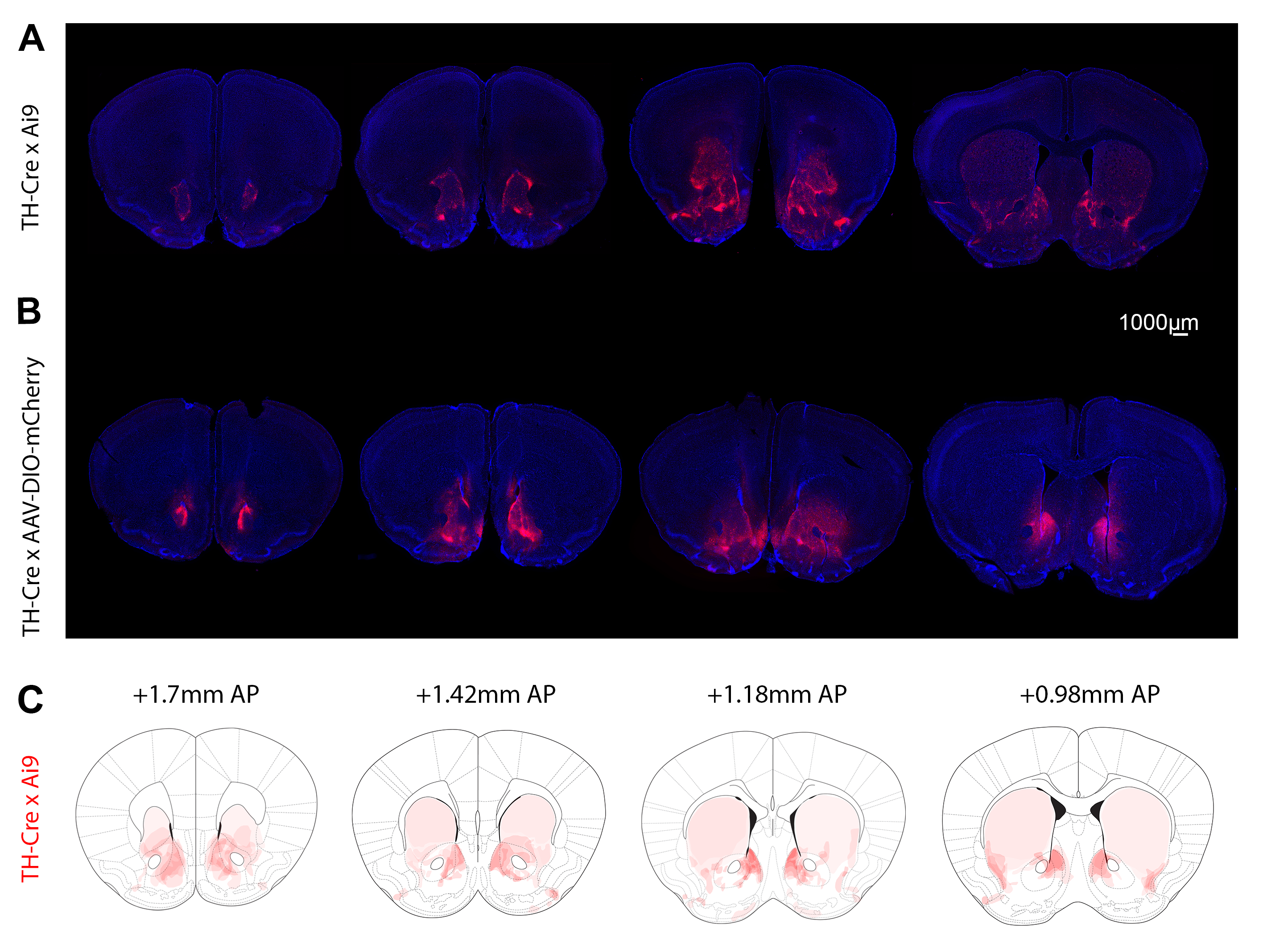

Supplement: Figure 3-2 — Characterization of NAc TH positive population. A. Representative images of td-Tomato positive cells in coronal sections spanning the NAc in TH-Cre x ai9 mice B. Representative images of viral targeting in coronal sections spanning the NAc in TH-Cre mice C. Representative diagrams of overall td-Tomato expression in coronal sections spanning the NAc of TH-Cre x ai9 mice. Download Figure 3-2, TIF file. [file eneuro-11-ENEURO.0487-24.2024-s004.tif]
